# A case of recurrent acute cholecystitis caused by *Actinomyces odontolyticus*, rare actinomycosis

**DOI:** 10.1186/s12879-022-07491-3

**Published:** 2022-06-04

**Authors:** Kento Furuya, Kenta Ito, Kyohei Sugiyama, Akitsugu Fujita, Hideyuki Kanemoto, Toshio Shimada

**Affiliations:** 1 Department of Clinical Laboratory Medicine, Shizuoka General Hospital, Kitaandou 4-27-1, Aoi-ku, Shizuoka, Japan; 2 Department of Pharmacy, Shizuoka General Hospital, Kitaandou 4-27-1, Aoi-ku, Shizuoka, Japan; 3 Department of Surgery, Shizuoka General Hospital, Kitaandou 4-27-1, Aoi-ku, Shizuoka, Japan

**Keywords:** *Actinomyces odontolyticus*, Cholecystitis, Gram stain, Ampicillin/sulbactam

## Abstract

**Backgrounds:**

*Actinomyces* species are gram-positive, obligate anaerobic rods and are rare causes of cholecystitis. Because *Actinomyces* species are anaerobic bacteria, it is difficult for *Actinomyces* to survive in bile apart from *A. naeslundii.* We experienced a case of recurrent acute cholecystitis caused by *A. odontolyticus.*

**Case presentation:**

A patient had been diagnosed with acute cholecystitis and treated one month before and after that, admitted to our hospital because of recurrent cholecystitis. Gram stain of the bile revealed gram-positive rods and gram-positive cocci. We found *A. odontolyticus* and MRSA in bile culture and MRSA in blood culture. We administered piperacillin-tazobactam and then changed it to ampicillin-sulbactam and vancomycin. The patient underwent laparoscopic cholecystectomy and was discharged safely.

**Conclusions:**

To our knowledge, this is the first case of cholecystitis caused by *A. odontolyticus*. Cholecystitis caused by *Actinomyces* species is rare. In addition, we may overlook it with the low positivity of bile cultures of *Actinomyces*. Whenever the cholecystitis recurs without any obstruction of the biliary tract, we should search for the gram-positive rods hidden in the bile, such as *A. odontolyticus,* as the causative organism, even if the bile culture is negative.

## Backgrounds

*Actinomyces* species are gram-positive, obligate anaerobic rods that colonize human’s upper respiratory tract, gastrointestinal tract, and female reproductive organs [1]. More than 30 species of *Actinomyces* have been identified to date and *A. israelii* is the most common pathogen [1].

In actinomycosis, intra-abdominal infections account for about 20%, and the most common site of infection is the ileum. There are few cases of cholecystitis due to *Actinomyces* species reported. To the best of our knowledge, our case must be the first case of cholecystitis caused by *A. odontolyticus.*

## Case presentation

**Fig. 1:**
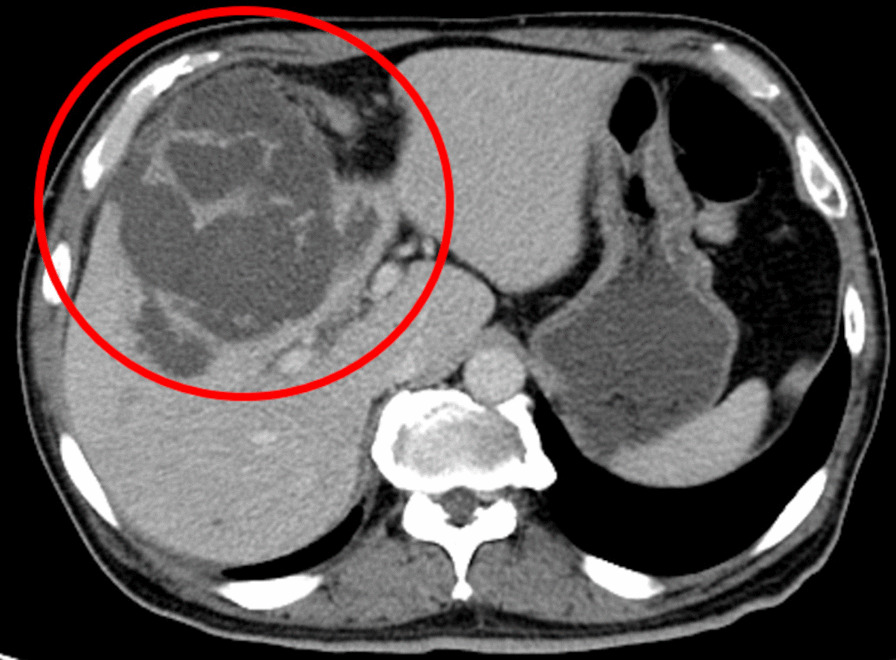
Contrast-enhanced abdominal CT demonstrated enlarged and multifocal gallbladder and abscesses around the gallbladder, some perforating into the right lobe of the liver and forming liver abscesses

**Fig. 2:**
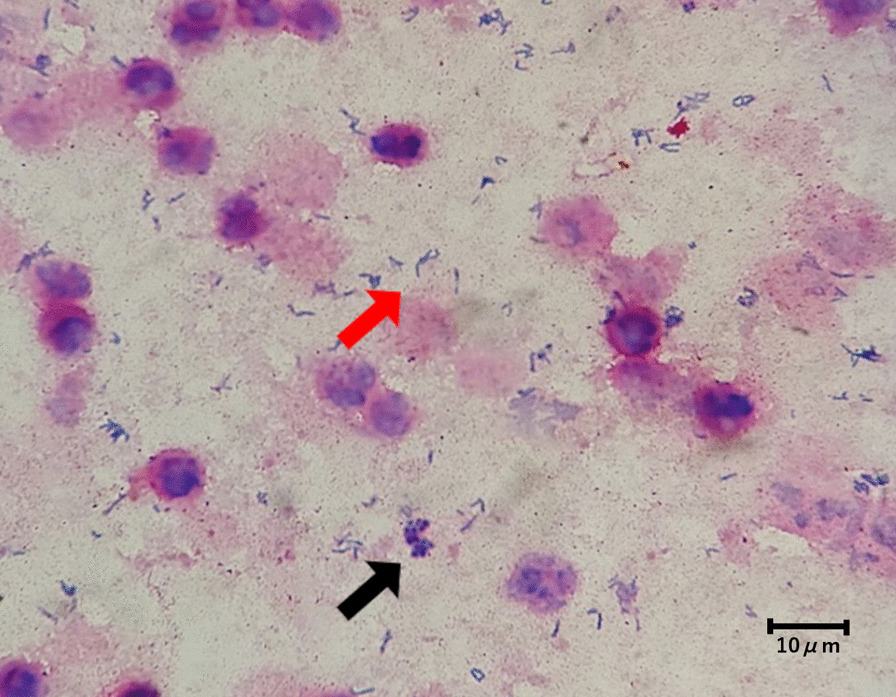
Gram stain of bile revealed gram-positive rods without an elongated radial pattern (red arrow) and gram-positive cocci (black arrow). Later, we identified gram-positive rods as *A. odontoliticus* and gram-positive cocci as MRSA (magnification × 1000, 300 dpi) (This image was acquired and captured using an Olympus BX51 microscope (Olympus, Japan) and Olympus DP20-5 (Olympus, Japan))

A 75-year-old Japanese man came to our hospital with fever, chills, and right hypochondrial pain. One and three years ago, this patient was already diagnosed twice with acute cholangitis and cholecystitis and then underwent endoscopic retrograde biliary drainage thirty-nine days before admission to our hospital. We demonstrated gram-positive rods, and gram-negative rods with Gram stain; namely *A. hydrophilia* and *E. faecalis* grew in the culture with no gram-positive rods. When the patient was transferred to our hospital, his body temperature was 39.9 °C, and he had tenderness in the right costal region. Blood tests showed white blood cell counts 23,700 /μL, C-reactive protein 23.2 mg/dL, aspartate aminotransferase 63 U/L, alanine aminotransferase 51 U/L, alkaline phosphatase 233 U/L, and γ-glutamyl transpeptidase 82 U/L. Contrast-enhanced CT of the abdomen showed an enlarged and multifocal gallbladder, intrahepatic perforation and abscesses around the gallbladder, some perforating into the right lobe of the liver and forming liver abscesses. But there was no obstruction of the biliary tract (Fig. 1). We diagnosed him with acute cholecystitis and liver abscess and then administered piperacillin/tazobactam. On the second day of admission, we decided to put cholecystectomy on hold to prioritize treatment of the liver abscess, and we implemented percutaneous transhepatic gallbladder drainage. Gram stain of the bile revealed gram-positive rods and gram-positive cocci (Fig. 2), which we identified as *A. odontolyticus* and MRSA. We also detected MRSA in the blood culture collected on the first day of admission. Judging from the culture results, we changed piperacillin/tazobactam to ampicillin/sulbactam and vancomycin. Blood cultures were negative on the fourth day of hospitalization. We performed laparoscopic cholecystectomy on the 14th day of hospitalization. The pathology of the gallbladder showed no evidence of malignancy. The patient got well. On the 20th day of hospitalization, we changed antibiotics to amoxicillin/clavulanate and linezolid, and after that, the patient was discharged safely. We administered antibiotics for a total of 5 weeks. After antibiotics were completed, he had no acute cholecystitis and liver abscess recurrence.

## Discussion and conclusion

*Actinomyces* species are gram-positive, obligate anaerobic bacteria that infect various parts of the human organs [2]. The most common site of *Actinomyces* infections is a cervicofacial area, and other locations include the central nervous system, chest, and pelvis. Hepatobiliary infection is rare and accounts for 5% of all Actinomyces infections [2].

Only 22 cases of cholecystitis caused by *Actinomyces* species have been reported in English literature (Table 1). As far as we know, this is the first case in which *A. odontolyticus* was the causative organism [3–15]. *A. naeslundii* was the most common causative organism seen in 7 cases [3–5], and *A. israelii* in 3 cases [6, 7]. The other 12 cases were pathologically diagnosed with Actinomycosis. These results were consistent with the fact that it was difficult for *Actinomyces* other than *A. naeslundii* to survive in an environment of bile salts [8]. In this case, we detected *A. odontolyticus* in bile cultures. We assume that coinfected MRSA consumed oxygen and created an anaerobic environment where *A. odontolyticus* could grow. When *A. naeslundii* was the causative organism, it was the single pathogen in 6 of 7 cases (85.7%) [3–5]. On the other hand, in the cases of *A. israelii*, 2 of 3 cases (66.7%) were infected with another bacteria; *Pseudomonas aeruginosa* was detected in one case [6], and *Pseudomonas aeruginosa* and *Haemophilus aprophilus* were seen in another case [7].

**Table 1:** Characteristics of the cholecystitis caused by *Actinomyces* species [3–15]

Number of cases—no.	22
Male:female	1:1
Average of age—year [range]	63.1 [41–86]
Gall stone—no. (%)	14 (63.6)
Liver abscess—no. (%)	2 (9.1)
Species—no.	
*A. naeslundii*	7
*A. islaelii*	3
Not identified	12
Gram stain positive—no	12
Bile culture positive—no	6
Past history of cholecystitis—no. (%)	6 (27.3)
Average of treatment duration—days [range]	109 [5–270]

Gram stain found gram-positive rods in 12 cases [8–10]. Of the 12 cases, six (50%) were positive for *Actinomyces* in culture [3, 10]. The mean age of the 22 patients was 63.1 years old, 14 cases (63.6%) had gallstones [3–6, 8, 10–14], and only 2 cases (9.1%) had a liver abscess [6, 15]. Of the 22 patients, six had a history of cholecystitis. Furthermore, one recurrent cholecystitis a few weeks after ending treatment was later proven to be caused by *Actinomyces*, as in our case [11]. Once the source of infection is controlled, the recommended treatment duration for acute cholecystitis is generally up to 7 days, even in severe cases [15]. However, in the case of cholecystitis caused by *Actinomyces*, the mean duration was 109 days in 22 patients because the appropriate duration of treatment for *Actinomyces* cholecystitis has not been established. Many cases underwent the treatment for long periods, like other actinomycosis such as lung infections.

Gram stain for *Actinomyces* visualizes and characterizes the presence of gram-positive rods with an elongated radial pattern [1]. However, as in another case of liver abscess caused by *A. odontolyticus* reported earlier [16], in this case, *A. odontolyticus* did not reveal a Gram stain with a typical elongated radial pattern. Thus, it may be difficult to distinguish *A. odontolyticus* from other gram-positive rods by Gram stain of bile. Furthermore, considering the low positive rate of bile culture in cholecystitis caused by *Actinomyces*, we may miss cholecystitis caused by *Actinomyces*. Even if the culture were negative, *Actinomyces* species should be considered the causative microorganism.

In conclusion, this is the first report of acute cholecystitis caused by *A. odontoliticus*. As cholecystitis related to *A. odontoliticus* is a rare condition, we may have been overlooked it because of the low positivity of bile culture and the absence of the typical elongated radial pattern on Gram stain. Furthermore, missing *Actinomyces* may be related to recurrent cholecystitis that may recall us *Actinomyces* infection.

## Data Availability

Not applicable.

## Abbreviations

MRSA: Methicillin‐resistant *Staphylococcus aureus*
CT: Computed tomography
